# Estimated pulse wave velocity can predict the incidence of new-onset atrial fibrillation: A 11-year prospective study in a Chinese population

**DOI:** 10.3389/fcvm.2022.912573

**Published:** 2022-08-22

**Authors:** Haojia Chen, Guanzhi Chen, Liling Zhang, Weiqiang Wu, Weijian Li, Xianxuan Wang, Xiuzhu Yan, Youren Chen, Shouling Wu

**Affiliations:** ^1^Shantou University Medical College, Shantou, China; ^2^Department of Cardiology, First Hospital of Medical College of Shantou University, Shantou, China; ^3^China Medical University, Shenyang, China; ^4^Department of Cardiology, Second Affiliated Hospital of Shantou University Medical College, Shantou, China; ^5^School of Foreign Language, Guangdong Polytechnic Normal University, Guangzhou, China; ^6^Department of Cardiology, Kailuan Hospital, North China University of Science and Technology, Tangshan, China

**Keywords:** estimated pulse wave velocity, atrial fibrillation, arterial stiffness, Chinese population, prospective cohort study

## Abstract

**Background:**

Arterial stiffness, a risk factor for atrial fibrillation (AF), is rarely applied in clinical practice because of the difficulty and high cost of its measurement. Estimated pulse wave velocity (ePWV) is a simple, reproducible, and non-invasive index of arterial stiffness. This study was to assess the predictive value of ePWV for the risk of new-onset AF.

**Methods:**

Subjects were selected from the Kailuan cohort study population who underwent initial physical examination between 2006 and 2008. A total of 96,561 subjects were ultimately included in the final analysis. ePWV was divided into four groups according to quartiles. The Kaplan–Meier method was used to calculate the cumulative incidence of AF. A Cox regression model was used to assess the predictive value of estimated arterial stiffness for new-onset AF.

**Results:**

Mean age of subjects was 51.47 ± 9.68 years, while 76,968 (79.65%) were male and 19,663 (20.35%) were female. During mean follow-up period of 11.77 years, 1,215 AF events occurred. Results of the Kaplan–Meier analysis showed that the incidence of new-onset AF increased with increase in ePWV. Cox regression analysis showed that in the total population, the incidence of new-onset AF was 1.64, 1.90, and 2.64 times higher in the medium, medium-high, and high ePWV groups, respectively, compared with the low ePWV group. When stratified according to sex, ePWV had higher predictive value in the female population.

**Conclusions:**

Increased ePWV increases the incidence of new-onset AF, and may promote application of more aggressive primary prevention.

**Trial registry name:**

Risk factors and intervention for cardiology, cerebrovascular and related disease (Kailuan Study); URL: http://www.chictr.org.cn/showproj.aspx?proj=8050; Registration number: ChiCTR-TNRC-11001489.

## Introduction

The prevalence of atrial fibrillation (AF) is increasing globally, with higher prevalence in developing countries, and an estimated age-adjusted prevalence of AF of 0.05 per 100 person-years in the Chinese population (1). The Global Burden of Cardiovascular Diseases, 2019, showed that AF is among the major global health burdens (2). More importantly, compared with non-AF patients, AF patients have a 5-fold risk of stroke, 1.95-fold risk of death, 4.62-fold risk of heart failure, and a higher risk of hospitalization, with 10–40% of AF patients being hospitalized annually (3–5). Unfortunately, although anticoagulant therapy may prevent strokes, AF patients may develop vascular dementia and cognitive decline, and have a poorer quality of life (3). Presently, regarding prediction of the risk of AF, clinical assessment is based on relevant AF risk factors (6), among which arterial stiffness is strongly associated with development of AF (7, 8). Additionally, carotid-femoral pulse wave velocity (cfPWV) is considered the gold standard for assessing arterial stiffness. Studies showed that cfPWV can be used to predict the onset of AF. Lin et al. (7) showed that for every 1-unit increase in cfPWV, the risk of developing AF increased 1.15-fold. Although cfPWV has good predictive value for the onset of AF, this index is relatively difficult and expensive to measure and is therefore usually unavailable (9). However, recent studies of subjects in the Paris population showed that estimated pulse wave velocity (ePWV) can be calculated using age and mean blood pressure (BP) in both healthy participants and in patients with essential hypertension without known cardiovascular diseases (CVD), using an equation generated by the Reference Values for Arterial Stiffness Collaboration, which predicts values similar to cfPWV (10). Using a large community-based cohort of 96,561 adults, we aimed to investigate whether ePWV predicts the risk of AF and if it can provide some guidance to clinicians for predicting the risk of AF in the Chinese population.

## Materials and methods

### Study participants

The Kailuan cohort study is an ongoing prospective cohort study (11, 12). Beginning from 2006 to 2008, a total of 11 hospitals, including Kailuan General Hospital and its affiliated hospitals, conducted physical examinations for active and retired employees from Tangshan Kailuan company once every 2 years, and collected relevant anthropometric and biochemical data. Each physical examination was conducted by medical staff who participated in the first examination and was performed at the same location for the same population in the same chronological order as the first examination. Additionally, the examined indices, anthropometric measurements, and biochemical indices were the same at each examination. Physical examination data were collected by uniformly trained professionals at each hospital and summarized at Kailuan General Hospital. The study followed the Declaration of Helsinki and was approved by the ethics committee of Kailuan General Hospital. All subjects signed an informed consent form. The epidemiological survey, anthropometric indicators, and biochemical indicators were tested and published previously (13, 14).

### Inclusion and exclusion criteria

The inclusion criteria were as follows: 1) subjects without AF who participated in the 2006–2008 physical examinations; and 2) consented to participate in the study and signed the informed consent form. The exclusion criteria were as follows: 1) history of cardiovascular or cerebrovascular events or tumors; 2) missing data on systolic blood pressure (SBP) or diastolic blood pressure (DBP) from the 2006 to 2008 physical examination; and 3) information on age missing from the 2006 to 2008 physical examinations.

### Measurement of BP

Subjects were prohibited from smoking, or drinking alcohol, tea, or coffee for 30 min before BP was measured. Subjects were asked to empty their bladders, remain calm, and sit quietly with their backs against a chair for 15 min. Subjects were then asked to take a sitting position in a comfortable environment for measurement of BP, with use of a table with a suitable height for the arm and with the right upper limb bare extended and 45 degrees abducted, and the elbow placed at the same level as the heart. A suitable cuff was selected based on the subject's upper arm circumference. The cuff was wrapped evenly against the skin on the upper arm so that the lower edge was approximately 2.5 cm above the elbow fossa, and the central air pocket of the cuff was located above the brachial artery. A calibrated bench-top mercury sphygmomanometer was used to measure the right brachial arterial pressure with a uniform slow deflation of 2–4 mmHg/s. SBP was defined as the first Korotkoff sound heard, while DBP was determined at the fifth phase. Both measurements were taken to the nearest 2 mmHg. The mean value of three BP measurements with 1-min intervals was taken as the final BP value of the subject.

### Biochemical measurements

Subjects were fasted for at least 8 h, and 5 ml of blood were drawn from the elbow vein between 7:00 am and 9:00 am on the day of the examination, and placed in a vacuum tube containing EDTA. Blood was centrifuged for 10 min at 3,000 rpm and room temperature (24°C), and serum in the upper phase was harvested for measurement within 4 h. Total cholesterol, low-density lipoprotein cholesterol and high-density lipoprotein cholesterol were measured using enzymatic methods (Mind Bioengineering Co. Ltd., Shanghai, China). Fasting glucose was measured using the hexokinase method.low-density lipoprotein cholesterol, and high-density lipoprotein cholesterol (7600 Auto matic Analyzer, Hitachi, Tokyo, Japan).

### Atrial fibrillation

An electrocardiograph (1350C Shanghai Optoelectronic Medical Electronic Instrument Co., Ltd., Shanghai, China) was used and 12-lead electrocardiogram was performed on subjects by a specialized physician according to conventional electrocardiographic criteria (15). Electrocardiographers at the Kailuan General Hospital reading center read and analyzed electrocardiograms (ECG) using the Minnesota ECG classification (16). AF was defined as diagnosis of AF by ECG during physical examination or hospitalization.

### Calculation of ePWV

The Reference Values for Arterial Stiffness Collaboration previously published equation that describe the correlation of cfPWV with age and mean blood pressure (MBP) in different a priori cardiovascular risk populations while considering non-linearities and interactions (17). Therefore, we used this equation to calculate ePWV: 7.84–0.33 × age + 3.8 × 10^−3^ × age^2^-1.97 × 10^−5^ × age^2^ × MBP + 2.5 × 10^−3^ × (age × MBP)−1.9 × 10^−3^ × MBP. MBP was calculated as diastolic BP+0.4 (SBP–DBP). Because there are no normal values for ePWV, we divided ePWV into four groups according to quartiles (low ePWV: ePWV ≤ 7.39; medium ePWV: 7.39 < ePWV ≤ 8.57; medium-high ePWV: 8.57 < ePWV ≤ 10.05; and high ePWV: ePWV > 10.05) (18).

### Measurement of brachial-ankle pulse wave velocity

Brachial-ankle pulse wave velocity (baPWV) was measured by a BP-203RPEIIIarteriosclerosis detection device [Omron Health & Medical (China) Co., Ltd., Dalian China]. The room temperature was kept at 22–25°C in the examination. The subjects were asked to lie flat on a bed quietly. The BP cuffs were applied to the four limbs, namely the upper arms and ankles of the lower limbs. The bare upper arms were wrapped by the cuffs with the cuff airbag marker against the brachial artery, and the lower cuff edge was 2–3 cm away from the elbow socket. The lower extremity cuff was located with the airbag on the inner side of the lower extremity, and the lower cuff edge was 1–2 cm away from the medial malleolus. The measurements should be carried out after a rest on the bed for at least 5 min. Every subject should take two measurements and the value of the second time as the final result. In total of 54,212 subjects were involved in the measurement of the baPWV test (19).

### Statistical methods

SAS 9.4 (SAS Institute, Cary, NC, USA) was used for data analysis. Continuous variables are presented as mean ± standard deviation, and one-way ANOVA was used for comparisons between different ePWV groups. Categorical variables are presented as n (%), and the χ^2^ test was used for comparisons between groups. The Kaplan–Meier method was used to calculate the cumulative incidence of AF, and the log-rank test was applied to test statistical differences. A Cox regression model was used to assess the effect of ePWV on new-onset AF. To reduce the effects of sex on the results of our analyses, subjects were divided into two subgroups according to sex for analysis. Given that the formula for calculation of ePWV is based on the European population, whether it can be applied to the Chinese population has yet to be clarified. Therefore, we performed a consistency test between ePWV and baPWV in the Kailuan population. Given the effect of hypertension and CVD on AF and arterial stiffness, we separately excluded patients with hypertension and CVD for sensitivity analyses. *P* < 0.05 was considered statistically significant (bilateral).

## Results

### General information on subjects

A total of 101,428 subjects participated in the 2006–2008 physical examinations. After excluding 3,380 cases with cardiovascular or cerebrovascular events, 330 cases with history of tumors, 1,157 cases missing data on SBP or DBP, and 0 cases missing data on age, 96,561 cases were included in the final analysis. Among them, 76,908 (79.65%) subjects were male and 19,653 (20.35%) subjects were female, with mean age of 51.47 ± 9.68 years. With increase in ePWV, the mean values of age, SBP, DBP, MBP, fasting blood glucose, and high-density lipoprotein cholesterol increased. Additionally, with increase in ePWV, the proportions of subjects who engaged in physical exercise, and who had hypertension, diabetes mellitus, or AF increased. The mean values of total cholesterol, low-density lipoprotein cholesterol, and body mass index, as well as the proportion of those who were drinkers of alcohol, were highest in the medium-high group (*P* < 0.05, Table 1).

**Table 1:** Characteristics of participants stratified by ePWV.

**Variable**	**Low ePWV** **≤ 7.39**	**Moderate** **(7.39, 8.57]**	**Moderate–** **high (8.57,** **10.05]**	**High** **ePWV >10.05**	***P***
Age, year	37.82 ± 8.45	48.33 ± 6.39	54.40 ± 6.29	65.39 ± 9.06	<0.01
Man, *n* (%)	16,508 (68.17)	19,335 (80.05)	20,088 (83.37)	20,977 (87.06)	<0.01
SBP, mm Hg	111.68 ± 10.93	123.65 ± 11.05	135.68 ± 14.02	151.71 ± 20.79	<0.01
DBP, mm Hg	73.81 ± 7.70	81.36 ± 7.53	87.02 ± 9.59	91.43 ± 13.14	<0.01
MBP, mm Hg	88.96 ± 8.35	98.27 ± 8.02	106.49 ± 10.27	115.54 ± 14.78	<0.01
Total Cholesterol, mg/dl	4.74 ± 1.05	4.98 ± 1.12	5.05 ± 1.19	5.02 ± 1.20	<0.01
Low-density lipoprotein cholesterol, mg/dl	2.31 ± 0.77	2.35 ± 0.86	2.37 ± 0.98	2.35 ± 1.02	<0.01
High-density lipoprotein cholesterol, mg/dl	1.48 ± 0.35	1.54 ± 0.39	1.57 ± 0.41	1.60 ± 0.45	<0.01
Fasting blood glucose, umol/L	5.13 ± 1.56	5.44 ± 1.59	5.61 ± 1.83	5.69 ± 1.93	<0.01
Body mass index, kg/m^2^	24.05 ± 3.46	25.04 ± 3.32	25.51 ± 3.37	25.50 ± 3.61	<0.01
ePWV, m/s	6.66 ± 0.51	7.98 ± 0.34	9.25 ± 0.42	11.57 ± 1.25	<0.01
Physical exercise, *n* (%)	1,841 (7.60)	2,412 (9.99)	4,083 (16.95)	5,708 (23.69)	<0.01
Hypertension, *n* (%)	993 (4.10)	6,192 (25.63)	14,789 (61.38)	19,580 (81.27)	<0.01
Diabetes Mellitus, *n* (%)	727 (3.00)	1,821 (7.54)	2,733 (11.34)	3,431 (14.24)	<0.01
AF, *n* (%)	48 (0.20)	151 (0.63)	264 (1.10)	752 (3.12)	<0.01
Drinking, *n* (%)	3,324 (13.80)	5,333 (22.08)	5,822 (24.16)	5,710 (23.70)	<0.01
Smoking, *n* (%)	7,946 (32.81)	9,273 (38.39)	8,865 (36.79)	7,892 (32.76)	<0.01

### The cumulative incidence of AF events in different ePWV groups

During the follow-up period of 11.77 ± 1.84 years, a total of 1,215 AF events occurred. The cumulative incidence of AF events increased gradually with increasing ePWV (*P* < 0.05, Table 2).

**Table 2:** Cumulative incidence of AF events in different ePWV groups.

**Group**	**Low**	**Moderate**	**Moderate–high**	**High**	**Total**	**Log-rank**
No. Of case	48	151	264	752	1,215	<0.01
Total	24,217	24,155	24,095	24,094	96,561	

### Cox proportional-hazards model analysis to assess predictive value of ePWV for AF

Cox regression analysis was conducted using the incidence of AF as the dependent variable, ePWV grouping as the independent variable, and the low ePWV group as the reference. Before correction, the risk of new-onset of AF was 3.16, 5.55, and 15.97 times higher in the medium, medium-high, and high ePWV groups, respectively. After further correction for age, sex, low-density lipoprotein cholesterol, high-density lipoprotein cholesterol, diabetes mellitus, body mass index, physical exercise, smoking, drinking, and hypertension, the risk of new-onset of AF was 1.64, 1.90, and 2.64 times higher in the medium, medium-high, and high ePWV groups, respectively (*P*trend < 0.05, Table 3). When grouped according to sex, the risk of new-onset of AF was 1.49, 1.72, and 2.31 times higher in male subjects and 2.55, 3.00, and 5.47 times higher in female subjects in the medium, medium-high, and high ePWV groups, respectively, compared with those in the low ePWV group after correcting for relevant confounders (*P*trend < 0.05, Table 3).

**Table 3:** Cox proportional-hazards model for AF among ePWV groups.

**Group**		**Model 1**	***P***	**Model 2**	***P***
Total	Low	1(Ref)		1(Ref)	
	Moderate	3.16 (2.28–4.37)	<0.01	1.64 (1.17–2.30)	<0.01
	Moderate–high	5.55 (4.08–7.55)	<0.01	1.90 (1.36–2.65)	<0.01
	High	15.97 (11.97–11.93)	<0.01	2.64 (1.83–3.80)	<0.01
	*P* trend	<0.01		<0.01	
Man	Low	1(Ref)		1(Ref)	
	Moderate	2.81 (1.96–4.05)	<0.01	1.49 (1.02–2.16)	<0.01
	Moderate–high	4.99 (3.54–7.04)	<0.01	1.72 (1.19–2.50)	<0.01
	High	14.13 (10.19–19.59)	<0.01	2.31 (1.54–3.45)	<0.01
	*P* trend	<0.01		<0.01	
Woman	Low	1 (Ref)		1 (Ref)	
	Moderate	4.16 (2.01–8.64)	<0.01	2.55 (1.18–5.48)	<0.01
	Moderate–high	6.56 (3.24–13.27)	<0.01	3.00 (1.34–6.73)	<0.01
	High	19.80 (10.26–38.23)	<0.01	5.47 (2.19–13.65)	<0.01
	*P* trend	<0.01		<0.01	

### The consistency checking between ePWV and baPWV

**Figure 1:**
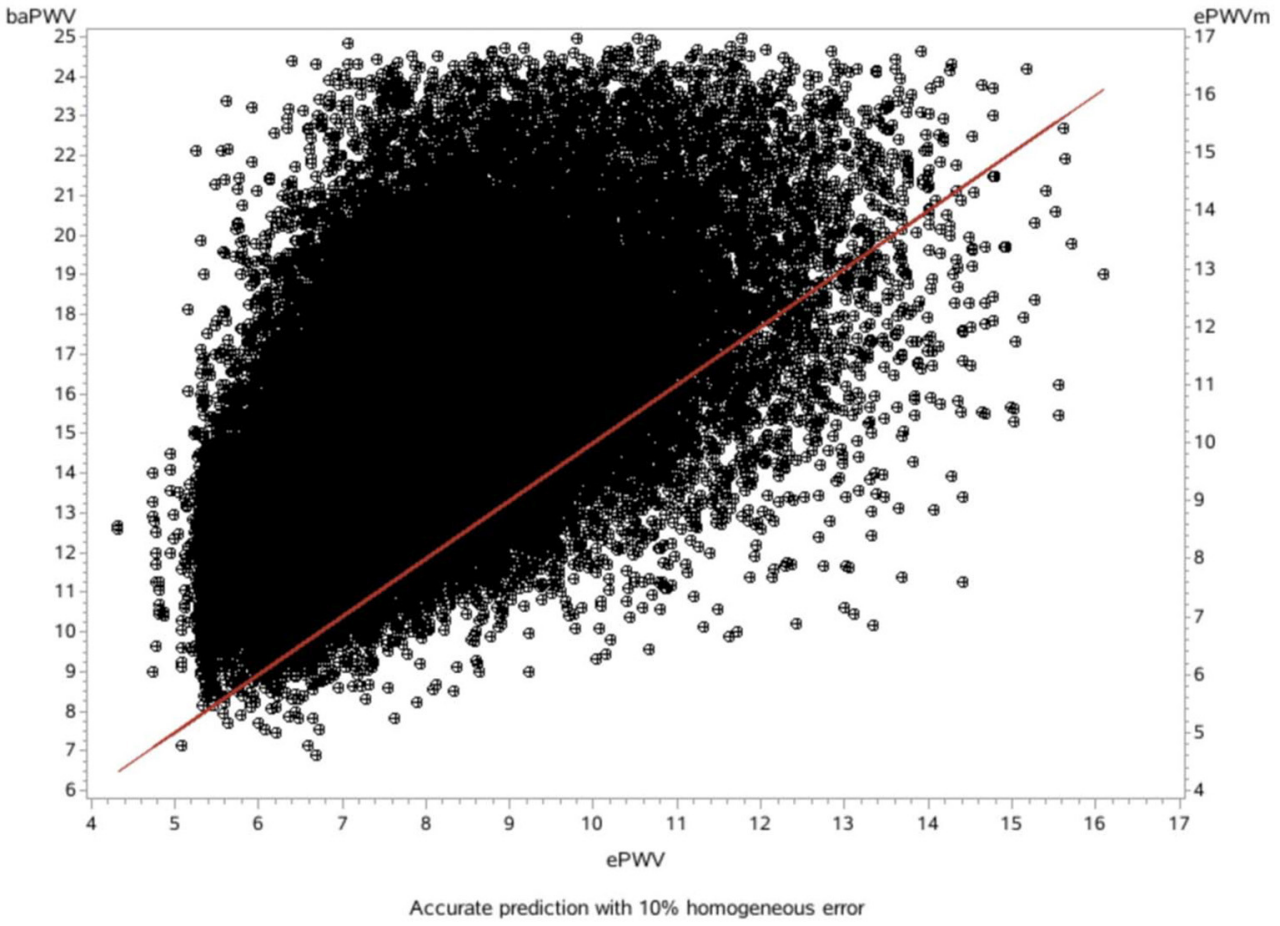
Scatter diagram showing the relationship between the baPWV and the ePWV (slope: 1.18, R^2^: 0.36).

**Figure 2:**
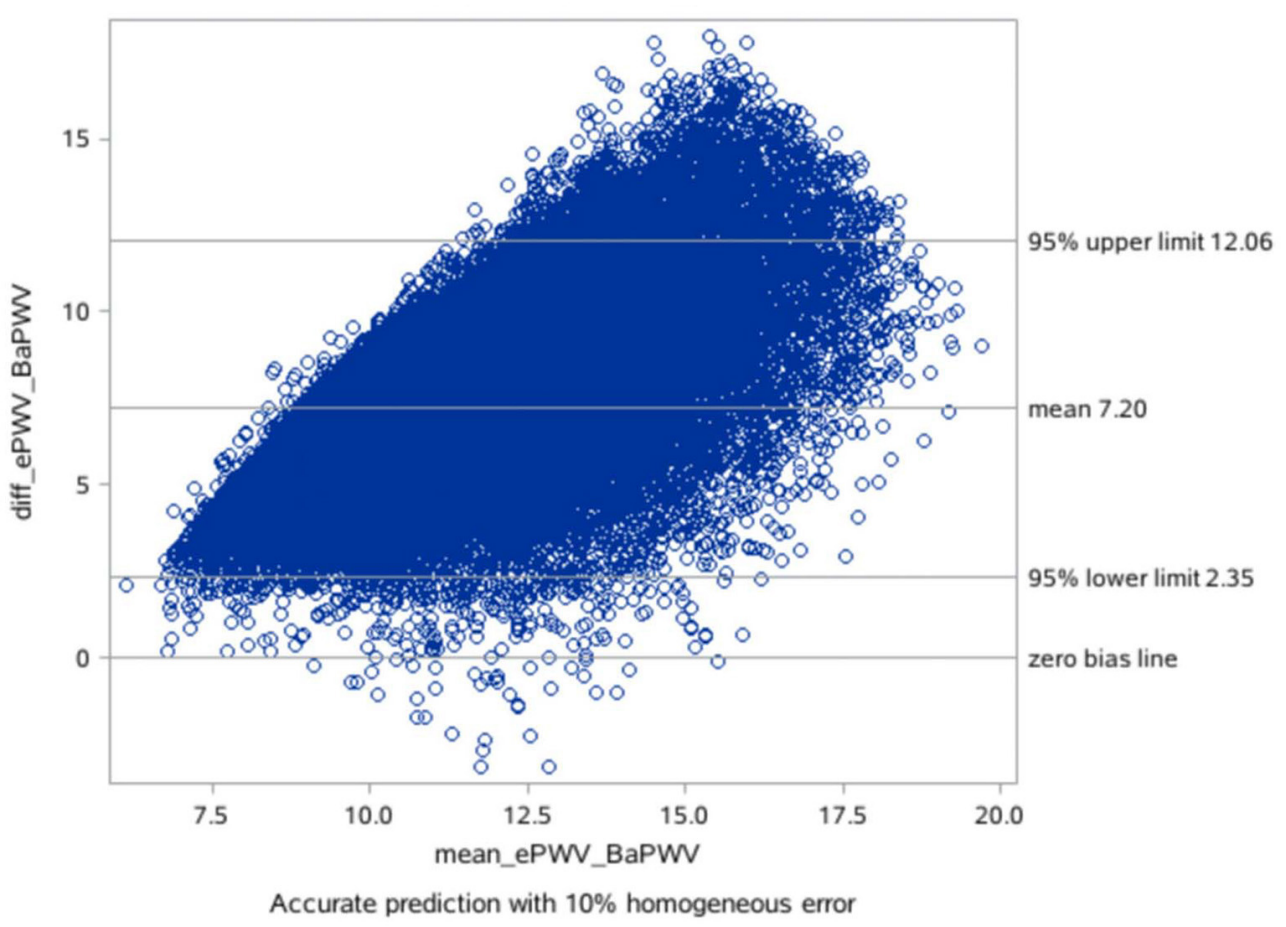
Bland Altman plot for the consistency analysis between the baPWV and the ePWV.

The mean difference between the measured baPWV and the ePWV was 7.20 m/s [95% LoA (limits of agreement): 2.35–12.06] as derived from the Bland-Altman plots (Table 4; Figures 1, 2).

**Table 4:** Comparison between the baPWV and the ePWV.

**Participants**	**baPWV, m/s**	**ePWV, m/s**	**Difference**	***P-*value for paired *t*-test**
Mean ± SD	15.01 ± 3.02	7.80 ± 1.58	7.20 ± 2.43	<0.001

### Sensitivity analysis

Sensitivity analysis was performed by separately excluding individuals with hypertension or CVD before the onset of AF. After correcting for relevant factors, the risk of new-onset of AF was 2.63, 3.70, and 5.13 times higher in the non-hypertensive population, and 1.65, 1.90, and 2.84 times higher in the population that excluded subjects with CVD in the medium, medium-high, and high ePWV groups, respectively, compared with the low ePWV group (*P*trend < 0.05, Table 5).

**Table 5:** Cox proportional-hazards model for AF among ePWV groups in different populations.

**Group**		**Model 1**	***P***	**Model 2**	***P***
Without hypertension	Low	1(Ref)		1(Ref)	
	Moderate	3.64 (2.51–5.27)	<0.01	2.63 (1.78–3.89)	<0.01
	Moderate–high	6.35 (4.46–9.04)	<0.01	3.70 (2.48–5.51)	<0.01
	High	12.11 (8.69–16.86)	<0.01	5.13 (3.27–8.07)	<0.01
	*P* trend	<0.01		<0.01	
Without CVD	Low	1(Ref)		1(Ref)	
	Moderate	3.23 (2.32–4.51)	<0.01	1.65 (1.17–2.33)	<0.01
	Moderate–high	5.73 (4.18–7.85)	<0.01	1.90 (1.35–2.68)	<0.01
	High	18.02 (13.38–24.28)	<0.01	2.84 (1.95–4.13)	<0.01
	*P* trend	<0.01		<0.01	

## Discussion

In the present study, we found that the incidence of AF increased with higher ePWV. The incidence of new-onset AF was 1.64, 1.90, and 2.64 times higher in the medium, medium-high, and high ePWV groups, respectively, compared with the low ePWV group. This suggests that elevated ePWV may predict new-onset AF. Our results are consistent with previous studies on measurements of arterial stiffness and the incidence of new-onset AF (7, 8, 19). According to a report based on the Atherosclerosis Risk in Communities Study (*n* = 13,907), the Multi-Ethnic Study of Atherosclerosis (*n* = 6,640), and the Rotterdam Study (*n* = 5,220), the risk of AF increased 1.15-fold and 1.12-fold for each 1-standard deviation increase in cfPWV and carotid intima-media thickness, respectively, in subjects from the three aforementioned study populations (7). In a study of 4,264 community populations, Cui et al. found that the incidence of new-onset AF in the highest of the augmentation index was 3.4 times higher than that of lowest tertiles (20). We found that the capacity of ePWV to predict risk of AF was slightly lower in men than in women. This may have been because of the higher aging index in women than men (21), as well as fewer associated risk factors for arterial stiffness and AF, resulting in a higher predictive value of ePWV for AF, which is also consistent with a lower incidence of AF in women compared with men (2) and with previous studies on arterial stiffness and AF (7).

Hypertension and occurrence of CVD before onset of AF may impact AF, and therefore we separately excluded these two populations in sensitivity analyses, and the results showed that ePWV remained a risk factor for AF. In the non-hypertensive population, the risk was significantly higher than that in the total population, suggesting that ePWV had a higher predictive value in the non-hypertensive population, which may have been because hypertension is a risk factor for arterial stiffness and development of AF (22–24), and thereby reduces the risk of ePWV in hypertensive populations. When we excluded subjects who developed CVD before the onset of AF, the risk was not significantly altered compared with the total population. This may have been because ePWV causes AF *via* mechanisms other than by leading to development of CVD.

On the basis of the correlation of cfPWV with age and MBP derived from analysis of a large database, ePWV is a convenient indicator of arterial stiffness. Previous studies have shown that ePWV and cfPWV are positively correlated (10). Previous studies have also reported that ePWV is a predictor of cardiovascular events independent of SCORE and Framingham Risk Score, and that its elevation is associated with subsequent mortality and the incidence of CVD (17, 18). However, it is premature to apply ePWV in clinical practice for predicting the risk of new-onset AF because current studies suggest ePWV shows strong cohort-dependence. Because previous studies on ePWV were based on European populations, further studies on ePWV and risk of new-onset AF are necessary. However, our consistency tests found that the mean difference between measured baPWV and ePWV was 7.20 m/s, and there was a good linear relationship and consistency between ePWV and baPWV in our study population. Moreover, our previous studies showed that ePWV correlated well with baPWV in the Chinese population, and ePWV was associated with cardiovascular disease CVD and all-cause mortality in the Chinese population (25). Therefore, use of the ePWV equation for predicting risk of AF in the Chinese population has positive implications.

The primary potential mechanisms underlying the causal association between arterial stiffness and AF are as follows: histological studies of atrial tissue confirmed the influence of atherosclerosis on progression of AF (26, 27). Moreover, atherosclerosis and its risk factors are associated with structural and electrical remodeling of the atria, while atrial fibrosis is a consequence of onset and progression of AF (28–30). In contrast, studies suggested that elevated atherosclerosis leads to left ventricular hypertrophy, impaired ventricular diastole, and left atrial enlargement, which in turn increases the risk of AF (31–34).

The present study had certain advantages. We investigated a large population (96,561 Chinese subjects) with continuous follow up of up to 11 years. Furthermore, this study has public health value and clinical significance: in non-AF populations, clinicians can easily, reproducibly, and non-invasively assess arterial stiffness and estimate the risk of subsequent AF simply by obtaining the patient's blood pressure and age, which can help reduce costs and injury from invasive testing, and may promote more aggressive primary prevention of AF at an early stage.

This study also had limitations. First, the study population was limited to employees of the Kailuan Group, most of whom live in communities in northern China, and regional differences were not considered. Therefore, the extent to which these findings can be applied to other populations remains unclear. However, the homogeneity of the Kailuan cohort may reduce potential confounding factors, and the large sample size is of certain guiding significance. Second, because the diagnosis of AF was based on ECG findings at physical examinations, hospitalization, or based on previous medical history, some individuals with paroxysmal AF may have gone unnoticed. However, this study has guiding significance for the Chinese population because the study population was followed up for up to 11.77 years. Third, previous studies on ePWV are based on European populations, and it is not clear whether they can be applied to the Chinese population. The correlation between ePWV and the risk of developing AF needs further clarification. Fourth, the risk factors associated with AF in the study population, such as smoking, alcohol consumption, and obesity, may change during the follow-up period, which may affect the risk assessment.

## Conclusions

Increased ePWV increases the incidence of new-onset AF, which may promote more aggressive primary prevention.

## Data availability statement

The data analyzed in this study is subject to the following licenses/restrictions: Datasets that were generated and analyzed in this study will not be published because of data protection, but the appropriate authors may have access to and/or analyze the datasets of the current study if reasonably required. Requests to access these datasets should be directed to SW: drwusl@163.com.

## Ethics statement

The studies involving human participants were reviewed and approved by [2006] No. 5 of Yilunzi. The patients/participants provided their written informed consent to participate in this study.

## Author contributions

HC, SW, and YC take responsibility for all aspects of the reliability and freedom from bias of the data presented and their discussed interpretation. HC, WL, and GC carried out the statistical analysis. LZ, WW, and XW participated in the study design. XY and SW strictly reviewed the manuscript. All authors read and approved the final draft of the manuscript.

## Funding

This work was supported by the National Natural Science Foundation of China (No. 81870312).

## Conflict of interest

The authors declare that the research was conducted in the absence of any commercial or financial relationships that could be construed as a potential conflict of interest.

## Publisher's note

All claims expressed in this article are solely those of the authors and do not necessarily represent those of their affiliated organizations, or those of the publisher, the editors and the reviewers. Any product that may be evaluated in this article, or claim that may be made by its manufacturer, is not guaranteed or endorsed by the publisher.

## Abbreviations

AF: atrial fibrillation
cfPWV: carotid-femoral pulse wave velocity
ePWV: estimated pulse wave velocity
BP: blood pressure
CVD: cardiovascular diseases
SBP: systolic blood pressure
DBP: diastolic blood pressure
ECG: electrocardiograms
MBP: mean blood pressure
baPWV: brachial-ankle pulse wave velocity.
